# Smartwatch-Based Blood Pressure Measurement Demonstrates Insufficient Accuracy

**DOI:** 10.3389/fcvm.2022.958212

**Published:** 2022-07-11

**Authors:** Maarten Falter, Martijn Scherrenberg, Karen Driesen, Zoë Pieters, Toshiki Kaihara, Linqi Xu, Enrico Gianluca Caiani, Paolo Castiglioni, Andrea Faini, Gianfranco Parati, Paul Dendale

**Affiliations:** ^1^Faculty of Medicine and Life Sciences, Hasselt University, Hasselt, Belgium; ^2^Heart Centre Hasselt, Jessa Hospital, Hasselt, Belgium; ^3^Department of Cardiology, Faculty of Medicine, KU Leuven, Leuven, Belgium; ^4^Faculty of Medicine and Health Sciences, Antwerp University, Antwerp, Belgium; ^5^Data Science Institute, Hasselt University, Hasselt, Belgium; ^6^Division of Cardiology, Department of Internal Medicine, St. Marianna University School of Medicine, Kawasaki, Japan; ^7^School of Nursing, Jilin University, Changchun, China; ^8^Department of Electronics, Information and Bioengineering, Politecnico di Milano, Milan, Italy; ^9^Institute of Electronics, Computer and Telecommunication Engineering, Consiglio Nazionale delle Ricerche, Milan, Italy; ^10^IRCCS Fondazione Don Carlo Gnocchi ONLUS, Milan, Italy; ^11^Department of Cardiovascular Neural and Metabolic Sciences, Istituto Auxologico Italiano, IRCCS, S. Luca Hospital, Milan, Italy; ^12^Department of Medicine and Surgery, University of Milano-Bicocca, Milan, Italy

**Keywords:** blood pressure, hypertension, smartwatch, Taffé method, digital health, cardiovascular disease, ambulatory blood pressure monitoring, blood pressure variability

## Abstract

**Background:**

Novel smartwatch-based cuffless blood pressure (BP) measuring devices are coming to market and receive FDA and CE labels. These devices are often insufficiently validated for clinical use. This study aims to investigate a recently CE-cleared smartwatch using cuffless BP measurement in a population with normotensive and hypertensive individuals scheduled for 24-h BP measurement.

**Methods:**

Patients that were scheduled for 24-h ambulatory blood pressure monitoring (ABPM) were recruited and received an additional Samsung Galaxy Watch Active 2 smartwatch for simultaneous BP measurement on their opposite arm. After calibration, patients were asked to measure as much as possible in a 24-h period. Manual activation of the smartwatch is necessary to measure the BP. Accuracy was calculated using sensitivity, specificity, positive and negative predictive values and ROC curves. Bland-Altman method and Taffé methods were used for bias and precision assessment. BP variability was calculated using average real variability, standard deviation and coefficient of variation.

**Results:**

Forty patients were included. Bland-Altman and Taffé methods demonstrated a proportional bias, in which low systolic BPs are overestimated, and high BPs are underestimated. Diastolic BPs were all overestimated, with increasing bias toward lower BPs. Sensitivity and specificity for detecting systolic and/or diastolic hypertension were 83 and 41%, respectively. ROC curves demonstrate an area under the curve (AUC) of 0.78 for systolic hypertension and of 0.93 for diastolic hypertension. BP variability was systematically higher in the ABPM measurements compared to the smartwatch measurements.

**Conclusion:**

This study demonstrates that the BP measurements by the Samsung Galaxy Watch Active 2 show a systematic bias toward a calibration point, overestimating low BPs and underestimating high BPs, when investigated in both normotensive and hypertensive patients. Standards for traditional non-invasive sphygmomanometers are not met, but these standards are not fully applicable to cuffless devices, emphasizing the urgent need for new standards for cuffless devices. The smartwatch-based BP measurement is not yet ready for clinical usage. Future studies are needed to further validate wearable devices, and also to demonstrate new possibilities of non-invasive, high-frequency BP monitoring.

## Introduction

Hypertension is a leading risk factor for heart disease (1). Current guidelines recommend to measure blood pressure (BP) using a validated auscultatory or (semi)automatic upper-arm sphygmomanometer (2, 3). While in the past BP measurement was mainly performed in the doctor’s office, nowadays it is known that the addition of out-of-office BP measurement provides better prognostic information than office BP measurement alone (4, 5). New evolutions in development of BP monitors aim to increase convenience while preserving accuracy (6).

Novel methods replace the oscillometric measurements of BP by cuffless measurements often using photoplethysmography (PPG), ballistocardiography (BCG) and/or electrocardiography (ECG) to estimate BP. This is performed by either analysing the pulse wave of the PPG signal (pulse wave analysis, PWA) (7), as is the case in the smartwatch that was used in this study, or by analysing a combination of PPG and ECG or BCG to calculate the pulse transit time (PTT) (8, 9). Other methods that are being investigated include the use of bio-impedance sensors for BP measurement (10). Advantages in using these methods are increased convenience as well as the possibility to easily increase the number of measurements per day, up to even continuous measurement. Other advantages are an increased role for the patient in managing their own health, possibly resulting in increased adherence to antihypertensive treatment (5).

While guidelines recommend only upper-arm inflatable cuff measurements of BP, commercial devices such as smartwatches capable of measuring BP using cuffless methods are already coming to market. In recent years, smartwatch devices first fulfilled the requirements for a medical device in the International Organization for Standardization (ISO) 81,060–2:2018 Non-invasive sphygmomanometers (5). However, it was recently emphasized that using these standards for new devices is inappropriate, and that new standards are needed (11). More recently, some devices were cleared by the United States Food and Drug Administration (FDA) and the European Union (Conformity Marketing, CE-label) (12). The Samsung Galaxy Watch Active 2 that was used in this study recently received CE-clearance, but algorithms and validation studies are not openly disclosed (8). In those studies about cuffless measurement that are openly available, devices mostly use data collected solely from normal healthy individuals (8). Because of the current scarce validation of the technology and low reliability of wrist BP measurement in general, the Korean Society of Hypertension – currently the only society that has published a position paper on smartwatch-based BP measurements - acknowledges the potential of smartwatch-based BP measurements for increasing awareness of BP and possibly to detect hypertension early in the general population, but is hesitant to recommend its use to monitor treatment response in hypertensive patients and patients with cardiovascular comorbidities (5).

In clinical practice immediate problems are now to be faced. One problem with unvalidated devices is that they cannot be actively used and prescribed before proper validation. A second and more pressing problem is that, once on the market, patients will use these devices for monitoring their health, and proper advice and interpretation by clinicians will be necessary. In a recent review, Elgendi et al. therefore emphasize that to increase reliability and validity of medical use of smartwatch-based BP measurements, more research is required in both normotensive and hypertensive subjects (8).

This study aims to investigate a recently CE-cleared smartwatch using cuffless BP measurement in a population with normotensive and hypertensive individuals scheduled for 24-h BP measurement. In doing so, this study aims to contribute to the evidence base for validating new technologies, already available on the consumer market, for medical use.

## Materials and Methods

### Design

The study is a prospective, single-arm, cross-sectional study.

### Participants

Consecutive patients that were scheduled for 24-h ambulatory blood pressure monitoring (ABPM) were recruited from the cardiology outpatient clinic. The inclusion criteria were as follows: scheduled for ABPM, age ≥ 18 years and able to understand the informed consent. The exclusion criteria were as follows: history of paroxysmal or permanent atrial fibrillation, difference in BP between both arms exceeding 20 mmHg as measured by a sphygmomanometer, inability to measure BP on one of both arms and inability to work with the smartwatch despite explanation by the research team. Demographic and medical information of the participants was collected from the hospital medical records and included age, gender, height, weight, cardiac risk factors, cardiac and non-cardiac comorbidities, and medication schedules. This study was approved by the ethics committee of Jessa Hospital and Hasselt University (file number 2021/050) and all subjects provided written informed consent.

### Measurement

Reference method measurements were performed using a validated automatic cuff-based upper-arm sphygmomanometer (Omron M2, Omron Corporation, Japan); ABPM was performed by a cuff-based, upper-arm automatic device (Mobil-O-Graph, IEM, Germany), measuring BP every 15 min throughout the day (8AM-11:59PM), and every 30 min throughout the night (00:00AM-7:59AM). A Samsung Galaxy Watch Active 2 (Samsung Electronics, South Korea) was used for cuffless BP measurement at the wrist (strap circumference range 50–70 mm) in combination with a Samsung Galaxy A21s smartphone (Samsung Electronics, South Korea). The smartwatch requires calibration to allow for BP measurement. The manufacturer recommends calibration at least every 28 days. Calibration was performed in all patients at inclusion. Algorithms that are used by the Samsung Galaxy Watch are not openly disclosed, but due to studies conducted by Samsung research and development institute it is likely that PWA using the PPG signal and derivatives in combination with a machine learning model are used (13).

Patients were first screened by measuring the BP consecutively at their left and right arms using the reference method. Smartwatches were then connected to the arm of the patient. ABPM devices are by standard protocol attached to the left arm; smartwatches were thus attached to the right arm in all patients. A calibration of the smartwatch was then performed according to the manufacturer’s instructions: three consecutive measurements were performed by the conventional BP monitor together with three measurements of the smartwatch.

Patients were instructed to manually start a measurement on the smartwatch each time the ABPM device had finished its measurement. This technique was chosen to avoid interference with the ABPM measurement. Patients were asked to perform as many measurements as possible in the 24-h interval, with a minimum of five measurements. The patients were instructed that during their sleep, measuring with the smartwatch would not be possible since a manual activation was necessary. They were instructed to resume measurements if they were awake at night or as soon as they woke up in the morning, after the first measurement of the ABPM. After 24 h, both the ABPM and the smartwatch were detached and read out.

### Statistical Analysis

Data analysis was performed using R [R Core Team (14), 2021] and SPSS (version 27, IBM Corporation). Descriptive statistics are given as means and standard deviations (SD) or numbers and percentages.

Bland-Altman plots were constructed and bias and limits of agreement were calculated. In the Bland-Altman plots difference is calculated as ABPM minus smartwatch measurements. However, as recently described by Taffé et al., the Bland-Altman method has certain limitations when comparing a new measurement method with a reference method (15). Also, the group of Bland and Altman has recently confirmed that the Bland-Altman method was never intended for calibration of a new device to a gold standard reference method (16). Taffé et al. therefore describe a new method for measuring bias and precision of new measuring devices, which is called the Taffé method. This method was used to calculate bias and precision. The Taffé method was carried out using R statistical software package MethodCompare. As current BP validation standards, as provided by the Association for Advancement of Medical Instrumentation (AAMI) and the European Society for Hypertension (ESH) (17, 18), rely on measures of the original Bland-Altman methodology, both the Bland-Altman and Taffé methods will be reported. While the nature of this study implies that the methodology for non-invasive sphygmomanometers (ISO 81060-2:2018) cannot be fully followed, reference criteria will be used to evaluate the results of this study. For the Bland-Altman analyses, all coupled smartwatch and ABPM measurements were used. For the Taffé method, all measurements for both devices were used as recommended by the authors.

Sensitivity, specificity, positive predictive value (PPV) and negative predictive value (NPV) were calculated for both systolic and diastolic BP during daytime. For these analyses the average systolic and diastolic BPs measured by the smartwatch during daytime was compared with the average ABPM daytime measurements as reference value. A cut-off for hypertension was based on the European Society of Cardiology (ESC) guidelines for measurement of daytime mean BP (≥135/85 mmHg) (1). Daytime was defined as 8 AM until midnight (as predefined in the ABPM). Receiver operating characteristic (ROC) curves were constructed and the Youden index was calculated to determine ideal cut-off points for diagnosing hypertension using the smartwatch device. Also, curves were constructed to visualize the optimal trade-off point for PPV and NPV for both systolic and diastolic BP. Again, for these analyses only daytime measurements were used, the ABPM measurement was used as a reference, and a cut-off of ≥135/85 mmHg was used as specified above.

BP variability was calculated using three widely used indices: the standard deviation (SD), the average real variability (ARV) and the coefficient of variation (CV) (19–21) which were calculated for both systolic and diastolic BP. SD and ARV were separately calculated for the daytime and night-time periods, and the weighted SD (SD_w_) was calculated as described by Parati et al. (21). CV was calculated for daytime BPs only. A paired-sample *t*-test was conducted to compare means, SDs, ARVs and CV, and the *p*-value is reported. *P*-values were not calculated if insufficient (≤2) night-time smartwatch measurements were available in each patient. A *p*-value of <0.05 was considered statistically significant. A comparison of daytime ARV per patient between smartwatch and ABPM was plotted.

Pulse pressure (difference of systolic minus diastolic BP) was calculated for each measurement of both devices; mean pulse pressure was calculated for the total population and means per patient were plotted.

### Outcomes

The primary outcome is the differential and proportional bias as defined by the Taffé method. It should be noted that due to lack in validation standards no cut-off can be pre-specified and a descriptive method will be used. Secondary outcomes include the Bland-Altman-based bias and precision as compared to ISO standards, AUC as defined by ROC analysis, and BPV as defined by ARV and SD.

## Results

### Patient Screening and Participation

A total of 40 patients participated in the study. A total of 101 patients was screened for participation. Seventeen patients were excluded based on the exclusion criteria (14 atrial fibrillation, 1 less than 18 years old, 1 could not understand the informed consent form, 1 with BP difference of >20 mmHg between both arms). Sixteen patients were not interested. Seven patients indicated that they were not able to work with the technology sufficiently. In 10 patients technical problems occurred: in 3 patients the watch was too large for making skin contact, in 5 patients the smartwatch was unable to perform a measurement due to reasons not specified by the smartwatch output, and two patients had a BP that was too high for calibration by the smartwatch resulting in an error message (191/94 mmHg and 216/120 mmHg). Eleven patients did not participate due to other reasons.

### Baseline Characteristics

Baseline characteristics and the mean number of measurements per device are depicted in Table 1.

**TABLE 1:** Baseline characteristics (*N* = 40).

Age (years)	57.7 ± 12.5
Gender (male)	23 (58)
BMI (kg/m^2^)	28.2 ± 4.6
Diabetes mellitus type 2	3 (8)
Hypertension (from history)	26 (65)
Current smoking	5 (13)
History of cardiovascular disease (ischemic heart disease, heart failure, arrhythmia, valvular heart disease and/or cardiomyopathy)	9 (23)
**Medication**	
ACE-inhibitor	10 (25)
Angiotensin receptor blocker	3 (8)
Calcium channel blocker	6 (15)
Thiazide and thiazide-like	6 (15)
Beta blocker	17 (43)
Loop diuretic	4 (10)
Mineralocorticoid receptor antagonist	0
Angiotensin receptor and neprilysin inhibitor	0
Aspirin	12 (30)
P2Y12 inhibitor	2 (5)
Mean 24-h systolic BP/diastolic BP (by ABPM, mm Hg)	130 ± 12/80 ± 10
**Average number of ABPM measures**	
24-h	76 ± 11
Daytime	62 ± 9
Night-time	14 ± 4
**Average number of smartwatch measures**	
24-h	31 ± 18
Daytime	28 ± 16
Night-time	3 ± 2
**Hypertension** (diagnosed by ABPM)	
Systolic hypertension (≥130 mmHg/24-h)	18 (45)
Diastolic hypertension (≥80 mmHg/24-h)	14 (35)
Systolic and/or diastolic hypertension	20 (50)

*ABPM, ambulatory blood pressure monitoring. ACE, angiotensin converting enzyme. BP, blood pressure. BMI, body mass index. SD, standard deviation. Daytime from 8AM to 11:59PM; night-time from midnight to 7:59 AM. Data as mean ± SD or n (%).*

### Bias and Precision Plots

#### Taffé Method

**FIGURE 1:**
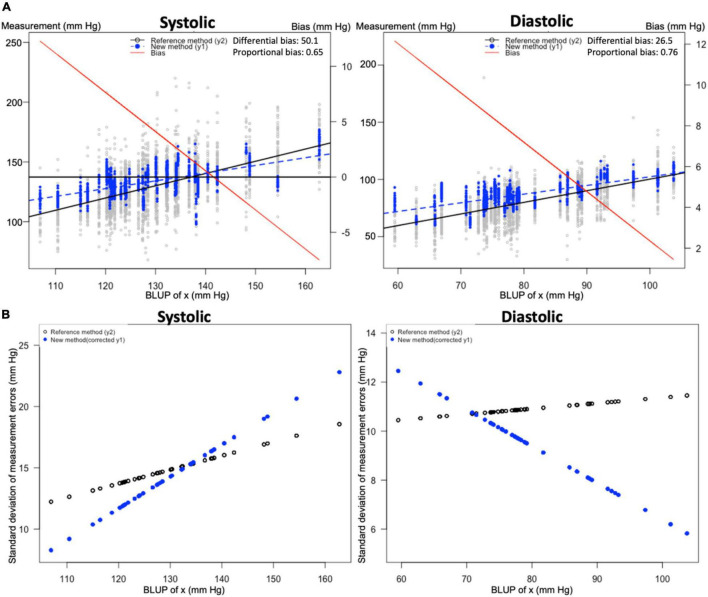
Bias and precision plots according to Taffé method. Figures were constructed according to the Taffé method. (A) Bias plots for systolic and diastolic blood pressure (BP). The *x*-axis indicates the best linear unbiased prediction (BLUP) or true value of BP. Smartwatch measurements (blue dashed lines) are compared to 24-h BP measurements (solid black line); both values are read from the leftmost *y*-axis. The bias (difference of smartwatch measurement compared to the 24-h BP measurement) is depicted as a solid red line; the value of bias is read from the rightmost *y*-axis. (B) Precision plots for systolic and diastolic blood pressure (BP). The *x*-axis indicates the best linear unbiased prediction (BLUP) or true value of BP. The standard deviation of measurement errors for both measurements are depicted with the 24-h BP measurement depicted in black and the smartwatch method depicted in blue.

Bias and precision plots according to the Taffé method are depicted in Figure 1. According to the Taffé method, bias and precision are non-constant measures and they depend on the estimated real BP (best linear unbiased prediction, BLUP).

For systolic BP, the smartwatch overestimates the BP up to a BP value of approximately 140 mmHg. After this point it underestimates the systolic BP, illustrating the presence of proportional (0.65, 95% confidence interval (CI) [0.60–0.70]) and differential (50.1, 95% CI [43.7–56.5]) bias. To further illustrate the implications of this proportional bias, it is clear from Figure 1 that at an estimated BP or BLUP of 112 mmHg, the bias is 10 mmHg; at a BLUP of 142 mmHg, the bias is 0 mmHg; and at a BLUP of 156 mmHg, the bias is −5 mmHg. Precision for the gold standard method was higher at higher BP values, while precision for smartwatch measurements was higher at lower BP values.

For diastolic BP, differential (26.5, 95% CI [23.3–29.8]) and proportional bias (0.76, 95% CI [0.72–0.80]) are present, resulting in an overestimation of the diastolic BP at all observed values, with a higher overestimation at lower values. Precision for the gold standard method was higher at lower BP values, while precision for smartwatch measurements was higher at higher BP values (from approximately 70 mmHg and upward).

#### Bland-Altman Method

Analysis according to the Bland-Altman method demonstrated an association between the true value and the measurement error, as also demonstrated by the Taffé method. As proposed by Bland and Altman (22), the appropriate next step is to log transform the data. This was performed but did not solve the problem of association. As a next step, Bland and Altman propose to use a regression approach, in which a linear regression is applied to obtain a regression line of the difference between measurements as a function of the average of the measurements (22).

**FIGURE 2:**
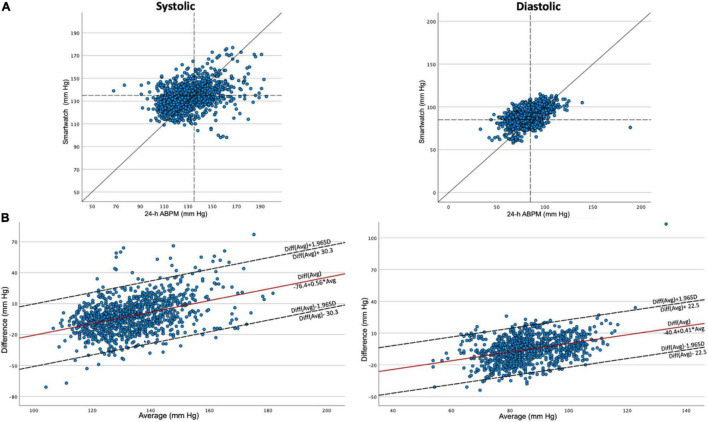
Scatterplots and Bland–Altman plots. Scatterplots (A) and Bland–Altman plots (B) for systolic and diastolic blood pressure measurements. In the scatterplots, cut-offs of 135 and 85 mm Hg are depicted as dashed lines. 24-h ABPM: 24-h ambulatory blood pressure monitoring. SD, standard deviation.

Scatterplots and Bland-Altman plots that were obtained through the method described above are depicted in Figure 2. Mean difference was −2.05 mmHg for systolic measurements and −5.58 mmHg for diastolic measurements, although because of the association between true value and measurement error as described above, these results should be interpreted with caution. For systolic measurements, the function for difference in function of average is described as: Diff = −76.4 + 0.56*Avg. The SD was 15.5. For diastolic measurements, the function for difference in function of average is described as: Diff = −40.4 + 0.41*Avg. The SD was 22.5.

### Accuracy Metrics

In Table 2 the sensitivity, specificity, PPV and NPV are depicted for detection of systolic, diastolic and both systolic and/or diastolic daytime hypertension by the smartwatch. Cut-off values of 135/85 mmHg were used for both the smartwatch and the ABPM measurements. For the detection of either systolic and/or diastolic hypertension, sensitivity was 83%, specificity was 41%, PPV was 54% and NPV was 75%. Analysis of these values using different cut-off values for the smartwatch is discussed in the following paragraph.

**TABLE 2:** Accuracy metrics of daytime smartwatch measures for the blood pressure cut-off of 135/85 mmHg.

	Systolic hypertension	Diastolic hypertension	Systolic and/or diastolic hypertension
Sensitivity (%)	71	92	83
Specificity (%)	73	52	41
PPV (%)	59	48	54
NPV (%)	83	93	75

*PPV, positive predictive value. NPV, negative predictive value.*

### Receiver Operating Characteristic Analysis and Positive Predictive Value/Negative Predictive Value Optimization

**FIGURE 3:**
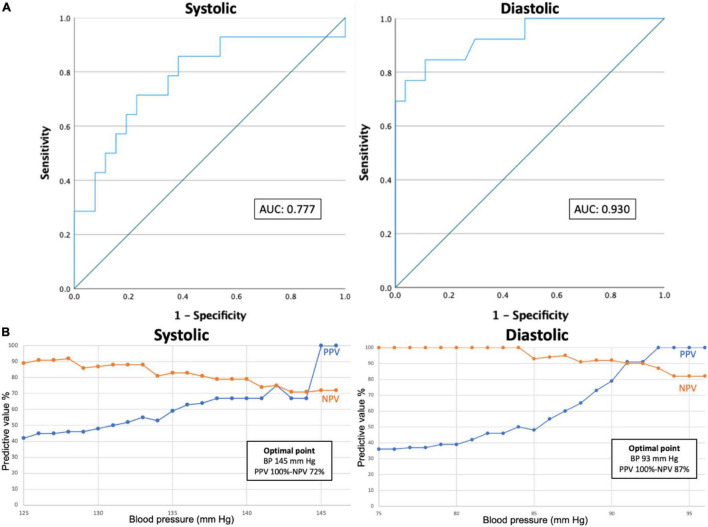
ROC curves **(A)** and PPV/NPV optimization curves **(B)**. ROC curves and PPV and NPV optimization curves in predicting systolic (left) or diastolic (right) hypertension. AUC, area under the curve. BP, blood pressure. PPV, positive predictive value. NPV, negative predictive value. ROC, receiver operating characteristic.

Receiver operating characteristic curves are depicted in Figure 3. For systolic BP the area under the curve (AUC) was 0.777 and the Youden index was 48.3 at a cut-off point of 136.1 mmHg. Sensitivity and specificity at this point were 71.4 and 76.9%, respectively. For diastolic BP AUC was 0.930 and the Youden index was 73.5 at a cut-off point of 89.9 mmHg. Sensitivity and specificity at this point were 84.6 and 88.9%, respectively.

Positive predictive value and NPV optimization curves were constructed. To obtain these curves, PPV and NPV were calculated for each cut-off ranging from 125 to 146 mmHg systolic and 75 to 96 mmHg diastolic. The optimal point was calculated as the point on which the sum of PPV and NPV was highest. For systolic BP, the optimal point was at 145 mmHg with a PPV and NPV of 100 and 72%, respectively. The highest NPV was obtained at 128 mmHg with a PPV and NPV of 46 and 92%, respectively. For diastolic BP, the optimal point was at 93 mmHg with a PPV and NPV of 100 and 87%, respectively. The highest NPV was reached at 84 mmHg with a PPV and NPV of 50 and 100%, respectively. The highest PPV was reached at 93 mmHg with a PPV and NPV of 100 and 87%, respectively.

### Blood Pressure Variability

**FIGURE 4:**
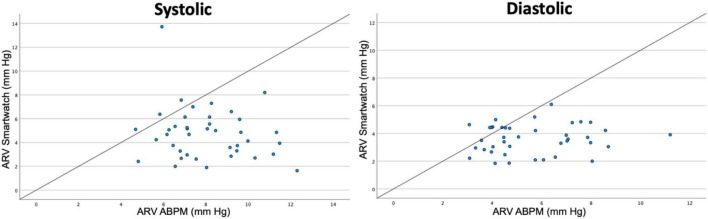
Scatterplots for blood pressure variability per patient based on daytime measurements. ARV, average real variability. ABPM, ambulatory blood pressure monitoring.

Blood pressure variability is depicted as daytime SD (SD_day_), SD_w_ and daytime ARV (ARV_day_) in Table 3. The results indicate that BP variability is higher in the ABPM measurements as compared to the smartwatch measurements. Paired sample *t*-tests for SD_day_ and ARV_day_ demonstrate a significant difference between BP variability of the ABPM versus the smartwatch measurements. Scatterplots of daytime ARV-values per patient are depicted in Figure 4, demonstrating a systematically higher BP variability in the ABPM measurements as compared to the smartwatch measurements. Furthermore, the CV was calculated. For SBP the CV was 3.6% ± 1.7% for the smartwatch and 11.4% ± 3.1% for the ABPM, *p* < 0.01; for DBP the CV was 4.2% ± 1.1% for the smartwatch as compared to 13.1% ± 3.8% for the ABPM device, *p* < 0.01, again confirming a significantly lower BP variability in the smartwatch measurements.

**TABLE 3:** Blood pressure mean and blood pressure variability: mean ± SD over *N* = 40 participants, with significance p of the difference between devices.

		Smartwatch	ABPM	*P*-value
M_day_ (mmHg)	Systolic	134 ± 11.7	132 ± 19.1	0.26
	Diastolic	88 ± 10.3	82 ± 14.8	< 0.001
SD_day_(mmHg)	Systolic	11.7 ± 2.0	19.1 ± 4.8	< 0.001
	Diastolic	10.3 ± 9.2	14.8 ± 3.1	< 0.001
SD_w_(mmHg)	Systolic	11.7 ± 1.3	18.8 ± 3.6	NA
	Diastolic	10.2 ± 0.8	14.6 ± 2.4	NA
ARV_day_ (mmHg)	Systolic	4.1 ± 2.2	11.0 ± 1.9	< 0.001
	Diastolic	3.3 ± 1.0	16.1 ± 1.9	< 0.001

*ABPM, ambulatory blood pressure monitoring. ARV_day_, average real variability during daytime. M_day_, mean daytime values. NA, not applicable. SD_day_, daytime standard deviation. SD_w_, weighted standard deviation. P-values were not calculated if insufficient (≤2) night-time smartwatch measurements were available in each patient.*

To clarify on possible bias due to the lower number of smartwatch measurements as compared to the ABPM, a full overview of BP variability parameters for all smartwatch measurements, all ABPM measurements and only the coupled ABPM measurements is given in Supplementary Table 1.

### Pulse Pressure

**FIGURE 5:**
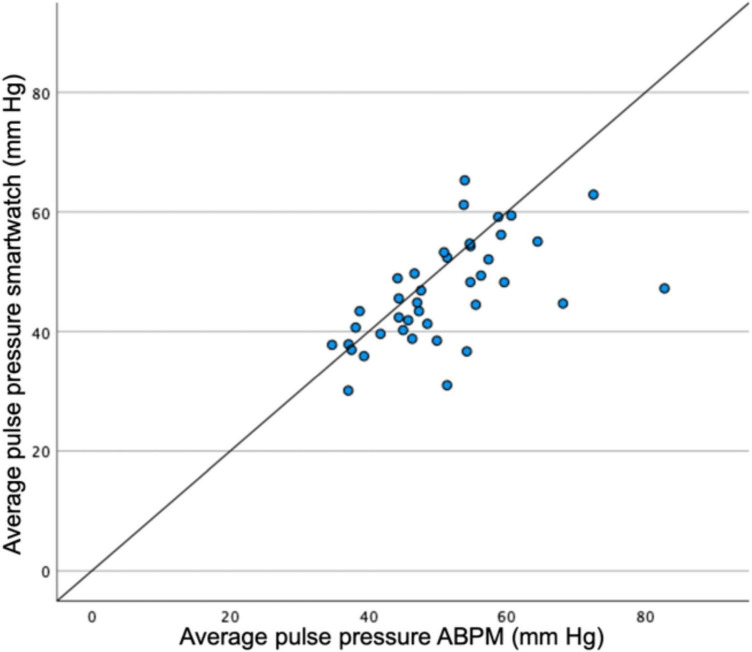
Scatterplot for pulse pressures measured by ABPM vs. smartwatch measurements per patient. ABPM, ambulatory blood pressure monitoring.

Pulse pressure (systolic BP minus diastolic BP) was compared for the total population and per patient. Mean pulse pressures over the total population were 49.2 mmHg and 45.6 mmHg for the ABPM and smartwatch measurements, respectively. The difference over the total population was thus 3.5 mmHg (*p* < 0.01). A scatterplot for mean pulse pressures per person is depicted in Figure 5.

## Discussion

To our knowledge, this is the first study comparing BP measured by a smartwatch to ABPM in a mixed normotensive and hypertensive population.

The main findings of this study are a proportional bias in the Taffé method, an association in the results needing correction in the Bland-Altman method and a BP variability that is systematically lower in the smartwatch as compared to the ABPM.

It should first be noted that of all 50 patients that were eligible for participation (i.e., all patients that did not meet an exclusion criterion, that were willing to participate and that were able to work with the technology) and that the device was tested on, 20% (10 patients) were not able to participate due to limitations of the technology (either not fitting, too high BP at calibration or unspecified error message). The fact that a high BP at calibration impedes the calibration process already limits the usage of the device in a hypertensive population. Also, 7 patients of the total screened population indicating not being able to work with the technology limits the usage in populations with low digital literacy.

It is known that validation standards for cuffless BP estimating devices are lacking, and the World Health Organization (WHO) and independent research groups have emphasized the urgent need for developing such validation standards (23, 24). With standards lacking, multiple analyses have been performed to provide as much insight as possible in the comparison of the two methods.

The results of the Taffé method indicate that, based on the bias plots, BP measurements are reliable in normal BP ranges. The farther BP deviates from a normal range, the larger the bias on the measurements becomes. Precision of both measurements is comparable in normal ranges. As already indicated by the developers of this method, Taffé et al., current validation methods suffer from the same limitations as the Bland-Altman analysis when comparing a new measurement method with a gold standard reference method and new international validation protocols using this new method are urgently needed (15). Existing validation protocols and criteria, such as the ISO 81060-2:2018 for non-invasive sphygmomanometers, are not applicable to this study as the smartwatch is a cuffless device in which a calibration step is required (11). Due to the fact that a calibration is necessary, providing three consecutive measurements as prescribed in the ISO protocol would not suffice to reliably validate the smartwatch. Still, it could be useful to compare the results of this study with the cut-offs that are proposed. While no 85 participants were included as proposed in the ISO standards, the amount of total paired measurements in this study (1,063) greatly exceeds the required 255 valid paired measurements. Criterion 1 of the ISO requirements states that for systolic and diastolic BP, mean difference is lower than or equal to ± 5 mmHg with a SD no greater than 8 mmHg (25). In our analysis the criterion for mean BP is met in systolic BP (−2.05) and is not met in diastolic BP (−5.58), and the criterion for SD is met in neither systolic (15.5) nor diastolic BP (22.5).

The ROC curves demonstrate acceptable AUCs (0.777 and 0.930 for systolic and diastolic BPs, respectively). For systolic BP maximal sensitivity and specificity were 71.4 and 76.9%, respectively at a BP of 136.1 mmHg and for diastolic BP maximal sensitivity and specificity were 84.6 and 88.9%, respectively at a BP of 89.9 mmHg. The PPV and NPV optimization curves demonstrate that high PPVs and NPVs can be achieved when using different cut-off points for the smartwatch compared to the reference standards. At first glance these results seem to be acceptable for a diagnostic test, but with validation standards lacking, one should keep questioning if these results are good enough to replace or complement gold standard BP measurements.

BP variability is systematically lower in the smartwatch compared to the ABPM measurement, with a systematic difference clearly being demonstrated in SD, SDw, ARV values, CV and in the ARV scatterplot.

Pulse pressure measurements are significantly different, but with a difference of 3.5 mmHg and no systematic error in the scatterplot, this latter result seems clinically acceptable.

Taking all these results together, namely the proportional bias in the Taffé method, the association needing correction in the Bland-Altman plots and the systematically lower BP variability, it seems that the smartwatch currently suffers from an anchoring point that is set when calibrating the device. After calibration, it tends to keep its BP measurements closer to the calibration point than the BPs are in reality, resulting in an overestimation of lower BPs, an underestimation of higher BPs, and thus a low BP variability. The results of our study thus indicate that a one-to-one translation of a conventional BP monitor to the smartwatch does not seem appropriate yet.

The Korean Society of Hypertension has stated that the future of cuffless devices might lie in detecting hypertension in the general population, but that they are currently not suited for monitoring treatment response in hypertensive patients (5). An additional limitation that is mentioned in their position paper is that accuracy of the smartwatch measurements may be further reduced in patients with aortic valve insufficiency, atrial fibrillation, peripheral vascular disease and other conditions affecting the cardiovascular system. The Korean Society does emphasize that, if accuracy is acceptable, smartwatch technology can increase hypertension awareness, especially in the younger population.

Other studies dare to broaden the scope of usage of these devices. Recent studies predict possibilities for integrating continuously measured BP measurements with other biological and environmental signals (26, 27). Data collected in this way can then be analyzed collectively through time-series analysis or machine learning techniques. This could in turn reveal more complex BP profiles in function of environmental conditions of the patient, or could even predict outcomes based on multiple parameters analyzed collectively. A recent review also broadens the scope to “anticipation medicine,” designed to identify increasing risk and predict the onset of cardiovascular events based on data collected over time (26). For these uses, a wearable device capable of measuring BP continuously including during the night would be most appropriate. Currently, the device used in this study is not capable of performing automatic measurements, but other wearables have been shown to perform accurate automatic measurements throughout the day (28).

At this moment, wearable cuffless BP monitors are not capable of replacing cuff-based BP monitors. If the technology is improved, it is possible that in the future there could be a role for complementary use next to cuff-based BP monitors, for example in different environments and settings, at home and away from home. Devices allowing automatic measurements can allow for continuous and nocturnal measurements, capabilities that conventional BP monitors do not offer.

To take the field forward several things are needed. First, there is an urgent need for validation standards for cuffless devices to allow for standardized research. Second, open disclosure of commercial validation studies could allow for better resource usage as studies will not have to be repeated unnecessarily. Third, further development of the cuffless devices will allow for new possibilities in the field of BP monitoring, especially unobtrusive, continuous and nocturnal monitoring. Finally, research should continue to focus on validation of these devices compared to conventional standards, but also on broadening its use and demonstrate new possibilities of continuous BP monitoring.

Patients will start using these devices today. It is thus up to researchers and clinicians to start understanding the vast range of possibilities that cuffless BP measuring has to offer.

### Limitations

This study has several limitations. First, this study uses a 24-h interval to conduct a validation study. As a smartwatch will be used in patients in a long-term setting a long-term validation study could be the scope of future research.

Second, while the algorithms that are used by the Samsung watch are not known, it is known that some algorithms use patient demographics (age, gender, and body mass index) to improve BP prediction based on PPG signals. As a single account was used on the Samsung watch, demographics were not adjusted in each patient (13).

Third, due to the nature of the intervention, patients had to manually activate the smartwatch after the measurement by the ABPM. This could result in bias by order effect which is a limitation of the study.

## Conclusion

This study demonstrates that the BP measurements by the Samsung Galaxy Watch Active 2 show a systematic bias toward a calibration point, overestimating low BPs and underestimating high BPs, when investigated in both normotensive and hypertensive patients. Standards for traditional non-invasive sphygmomanometers are not met, but these standards are not fully applicable to cuffless devices, emphasizing the urgent need for new standards for cuffless devices.

The smartwatch-based BP measurement is not yet ready for clinical usage. However, when used complementary to a cuff-based BP monitor, BP measurements might be of value. Future studies are needed to further validate wearable devices, and also to demonstrate new possibilities of non-invasive, high-frequency BP monitoring.

## Data Availability Statement

The raw data supporting the conclusions of this article will be made available by the authors, without undue reservation.

## Ethics Statement

The studies involving human participants were reviewed and approved by the Ethische Toetsingscommissie Jessa Ziekenhuis. The patients/participants provided their written informed consent to participate in this study.

## Author Contributions

MF: conceptualization, experiment conduction, data analysis, and manuscript writing and editing. MS: conceptualization and manuscript writing and editing. KD: experiment conduction and data handling. ZP: data analysis. TK: conceptualization and writing – review and editing. LX: writing – review and editing. EC, PC, and AF: data analysis and writing – review and editing. GP: writing – review and editing and supervision. PD: conceptualization, writing – review and editing, resources, and supervision. All authors contributed to the article and approved the submitted version.

## Conflict of Interest

The authors declare that the research was conducted in the absence of any commercial or financial relationships that could be construed as a potential conflict of interest.

## Publisher’s Note

All claims expressed in this article are solely those of the authors and do not necessarily represent those of their affiliated organizations, or those of the publisher, the editors and the reviewers. Any product that may be evaluated in this article, or claim that may be made by its manufacturer, is not guaranteed or endorsed by the publisher.

## Supplementary Material

Supplementary Results Blood samples were available for 15 out of 40 patients. Two patients (27%) had chronic kidney disease as defined by an estimated glomerular filtration rate (eGFR) of <60 mL/min/1.73 m^2^. None of these patients had an eGFR of <45 mL/min/1.73 m^2^.
