# Supplementary material for: Smartwatch-Based Blood Pressure Measurement Demonstrates Insufficient Accuracy
Source: Front Cardiovasc Med. 2022 Jul 11;9:958212. doi: 10.3389/fcvm.2022.958212 (PMC9309348; doi:10.3389/fcvm.2022.958212)
Supplement: Supplementary Results — Blood samples were available for 15 out of 40 patients. Two patients (27%) had chronic kidney disease as defined by an estimated glomerular filtration rate (eGFR) of <60 mL/min/1.73 m2. None of these patients had an eGFR of <45 mL/min/1.73 m2. [file Table_1.docx]

Supplementary Files

**Supplementary Table 1 – Blood pressure mean and blood pressure variability: mean ± SD over N=40 participants, with significance p of the difference between devices**

|  |  | Smartwatch | ABPM-c | P-value (ABPM-c vs smartwatch) | ABPM | P-value (ABPM vs smartwatch) |
| --- | --- | --- | --- | --- | --- | --- |
| **M_day_** (mmHg) | **Systolic** | 134 | 132 | 0.012 | 132 | 0.26 |
|  | **Diastolic** | 88 | 83 | <0.001 | 82 | <0.001 |
| **M_night_** (mmHg) | **Systolic** | 133 | 123 | NA | 119 | NA |
|  | **Diastolic** | 89 | 78 | NA | 71 | NA |
| **SD_w_**(mmHg) | **Systolic** | 11.7 ± 1.3 | 17.2 ± 3.4 | NA | 18.8 ± 3.6 | NA |
|  | **Diastolic** | 10.2 ± 0.8 | 13.9 ± 2.4 | NA | 14.6 ± 2.4 | NA |
| **SD_day_**(mmHg) | **Systolic** | 11.7 ± 2.0 | 17.4 ± 4.8 | <0.001 | 19.1 ± 4.8 | <0.001 |
|  | **Diastolic** | 10.3 ± 9.2 | 14.0 ± 3.1 | <0.001 | 14.8 ± 3.1 | <0.001 |
| **SD_night_**(mmHg) | **Systolic** | 11.5 ± 2.0 | 14.6 ± 7.3 | NA | 17.6 ± 4.1 | NA |
|  | **Diastolic** | 9.6 ± 1.7 | 12.8 ± 4.2 | NA | 14.1 ± 3.0 | NA |
| **ARV_day_** (mmHg) | **Systolic** | 4.1 ± 2.2 | 11.8 ± 3.7 | <0.001 | 11.0 ± 1.9 | <0.001 |
|  | **Diastolic** | 3.3 ± 1.0 | 6.3 ± 4.5 | <0.001 | 16.1 ± 1.9 | <0.001 |
| **ARV_night_** (mmHg) | **Systolic** | NA | NA | NA | 10.3 ± 3.4 | NA |
|  | **Diastolic** | NA | NA | NA | 3.7 ± 2.2 | NA |

Depicted are smartwatch measurements compared to the coupled measurements of the ABPM (ABPM-c) and all measurements by the ABPM (ABPM).

*ABPM: ambulatory blood pressure monitoring. ABPM-c: ambulatory blood pressure monitoring, only coupled measurements. ARV_day_: average real variability during daytime. M_day_: mean daytime values. M_night_: mean nighttime values. NA: not applicable. SD_w_: weighted standard deviation. SD_day_: daytime standard deviation. SD_night_: nighttime standard deviation. P values comparing nighttime measures were not calculated because the number of Smartwatch measurements at night was ≤2 in some participants.*
